# Impaired cardiac and peripheral hemodynamic responses to inhaled β_2_-agonist in cystic fibrosis

**DOI:** 10.1186/s12931-015-0270-y

**Published:** 2015-09-05

**Authors:** Erik H. Van Iterson, Stephen R. Karpen, Sarah E. Baker, Courtney M. Wheatley, Wayne J. Morgan, Eric M. Snyder

**Affiliations:** School of Kinesiology, University of Minnesota, Cooke Hall, 1900 University Ave SE., Minneapolis, MN 55455 USA; College of Pharmacy, University of Arizona, 1295 N Martin Ave, Tucson, AZ 85721 USA; Department of Anesthesiology, Mayo Clinic, 200 First Street SW, Rochester, MN 55905 USA; Department of Cardiovascular Diseases, Mayo Clinic, 200 First Street SW, Rochester, MN 55905 USA; Department of Pediatrics, University of Arizona, 1501 N. Campbell Avenue, Room 3301, Tucson, AZ 85724 USA

## Abstract

**Background:**

Pulmonary system dysfunction is a hallmark of cystic fibrosis (CF) disease. In addition to impaired cystic fibrosis transmembrane conductance regulator protein, dysfunctional β_2_-adrenergic receptors (β_2_AR) contribute to low airway function in CF. Recent observations suggest CF may also be associated with impaired cardiac function that is demonstrated by attenuated cardiac output (Q), stroke volume (SV), and cardiac power (CP) at both rest and during exercise. However, β_2_AR regulation of cardiac and peripheral vascular tissue, *in-vivo*, is unknown in CF. We have previously demonstrated that the administration of an inhaled β-agonist increases SV and Q while also decreasing SVR in healthy individuals. Therefore, we aimed to assess cardiac and peripheral hemodynamic responses to the selective β_2_AR agonist albuterol in individuals with CF.

**Methods:**

18 CF and 30 control (CTL) subjects participated (ages 22 ± 2 versus 27 ± 2 and BSA = 1.7 ± 0.1 versus 1.8 ± 0.0 m^2^, both *p* < 0.05). We assessed the following at baseline and at 30- and 60-minutes following nebulized albuterol (2.5mg diluted in 3.0mL of normal saline) inhalation: 12-lead ECG for HR, manual sphygmomanometry for systolic and diastolic blood pressure (SBP and DBP, respectively), acetylene rebreathe for Q and SV. We calculated MAP = DBP + 1/3(SBP–DBP); systemic vascular resistance (SVR) = (MAP/Q)•80; CP = Q•MAP; stroke work (SW) = SV•MAP; reserve (%change baseline to 30- or 60-minutes). Hemodynamics were indexed to BSA (Q_I_, SV_I_, SW_I_, CP_I_, SVR_I_).

**Results:**

At baseline, CF demonstrated lower SV, SV_I_, SW, and SW_I_ but higher HR than CTL (*p* < 0.05); other measures did not differ. At 30-minutes, CF demonstrated higher HR and SVR_I_, but lower Q, SV, SV_I_, CP, CP_I_, SW, and SW_I_ versus CTL (*p* < 0.05). At 60-minutes, CF demonstrated higher HR, SVR, and SVR_I_, whereas all cardiac hemodynamics were lower than CTL (*p* < 0.05). Reserves of CP, SW, and SVR were lower in CF versus CTL at both 30 and 60-minutes (*p* < 0.05).

**Conclusions:**

Cardiac and peripheral hemodynamic responsiveness to acute β_2_AR stimulation via albuterol is attenuated in individuals with CF, suggesting β_2_AR located in cardiac and peripheral vascular tissue may be dysfunctional in this population.

## Background

A well-recognized phenotype of cystic fibrosis (CF) disease includes signs and symptoms of chronic sinusitis and pulmonary airway dysfunction, which are strong predictors of morbidity and mortality in individuals with this disease [1–6]. Primary structural and physiological changes that occur within the pulmonary system, including low airway surface fluid, attenuated mucociliary clearance, and airway obstruction are caused by mechanisms that impair chloride (Cl^−^) and sodium (Na^+^) regulation specifically related to malfunctioning or absent cystic fibrosis transmembrane conductance regulator (CFTR) protein, and impaired epithelial Na^+^ channel (ENaC) inhibition [2, 7–10]. Structural and physiological abnormalities of the pulmonary system in CF disease readily transgress to functional impairments as assessed by airway and gas-transfer tests. Low forced expiratory volume in one second (FEV_1_), attenuated capacity of gas transfer within lungs, and reduced oxygen uptake (VO_2_) at rest and during exercise are leading pulmonary system abnormalities in CF [4, 6, 8, 9, 11, 12]; which, more importantly, strongly relate with morbidity and mortality in this population [1–3, 5, 6, 12].

Important evidence is accumulating that suggests certain individuals with CF demonstrate impaired β_2_-adrenergic receptor (β_2_AR) function in pulmonary airways [13–19]. Blunted resting pulmonary airway function in addition to attenuated airway responsiveness following inhalation of the selective β_2_AR agonist albuterol may be linked to dysfunction of airway β_2_AR in CF [13, 14]. In fact, Marson et al. further suggests that impaired spirometry responses following inhalation of albuterol indicates disease severity in CF [14].

In this context and taking into account that β_2_AR are found in abundant concentrations in pulmonary as well as cardiac and systemic vascular tissue [13–20]; observations relating pulmonary airway dysfunction to impaired airway β_2_AR in CF are noteworthy and may have critical clinical implications in this population [13, 14, 19]. As such, our group and others have shown that individuals with CF demonstrate attenuated cardiac output (Q), large arterial dysfunction, abnormal ventilatory mechanics, and attenuated lung gas-transfer [8, 9, 11, 12, 21]. Cumulatively, these observations suggest that there is global cardiovascular system impairment in individuals with CF; and, that the hallmark clinical phenotype of this disease is not isolated to pulmonary system dysfunction.

Accordingly, because we have previously demonstrated that inhalation of albuterol evokes increases in Q and stroke volume (SV) that accompany decreases in systemic vascular resistance (SVR) in healthy individuals [22], the aim of the present study was to assess cardiac and peripheral vascular function in response to acute inhalation of the β_2_AR selective-agonist albuterol in order to enhance our understanding of whether pathways related to β_2_AR in cardiac and peripheral vascular tissue are impaired, and associated with attenuated cardiac and peripheral vascular hemodynamic function in individuals with CF.

## Methods

### Participants

Forty-eight adults participated in this cross-sectional study (CF = 18; healthy individuals = 30 [controls]) (characteristics, Table 1). Cystic fibrosis in individuals was confirmed by a positive sweat test (≥60.0 millimole/liter Cl^−^) and genotyping of the ∆F508 mutation of CFTR. Individuals with CF who met the following exclusion criteria were not enrolled due to safety concerns: experienced a pulmonary exacerbation within the last two weeks or pulmonary hemorrhage within six months resulting in greater than 50 cc of blood in the sputum, were taking any antibiotics for pulmonary exacerbation, or if they were taking any experimental drugs related to CF. The Arizona Respiratory Center and its affiliated CF clinic at the University of Arizona Medical Center were used to recruit all individuals with CF. Word of mouth and posted advertising around the University of Arizona were used to recruit control participants. The protocol was reviewed and approved by the University of Arizona Institutional Review Board. All participants provided written informed consent prior to study, and all aspects of the study were performed according to the Declaration of Helsinki.

**Table 1:** Participant characteristics

	Control	Cystic fibrosis	*P*
n	30	18	
Age, years	27±2	22±2	0.04
Gender, male/female	18/12	13/5	0.54
Height, cm	172±2	167±2	0.07
Weight, kg	71±2	64±4	0.09
Body mass index, kg/m^2^	24±1	23±1	0.32
Body surface area, m^2^	1.8±0.0	1.7±0.1	0.04
Blood urea nitrogen, mg/dL	14.4±0.8	15.1±1.1	0.61
Serum creatinine, mg/dL	1.01±0.03	0.92±0.04	0.18
Serum sodium, mEq/L	139±0	138±1	0.11
Serum chloride, mEq/L	105±0	103±1	0.20
Albuterol dose (body weight standardized)			
Mean, μg/kg	36±1.2	41.0±2.0	0.04
Functional capacity and physical activity			
Peak oxygen uptake, mL/kg/min	35.1±2.1	23.7±2.2	<0.01
Peak oxygen uptake, % of predicted	97±6	55±5	<0.01
Exercise hours/week (self report)	7±1	9±3	0.23
Pulmonary function test parameters			
Forced vital capacity (L)	4.6±0.2	3.7±0.3	0.01
Forced vital capacity (%predicted)	97.3±2.8	83.2±4.7	0.02
FEV_1_ (L)	3.7±0.1	2.7±0.3	<0.01
FEV_1_ (%predicted)	94.7±2.6	73.8±6.1	<0.01
FEV_1_/FVC	0.8±0.0	0.7±0.0	<0.01
FEF_25-75_ (L/sec)	3.7±0.1	2.2±0.4	<0.01
FEF_25-75_ (%predicted)	89.6±4.0	54.2±8	<0.01
Medications (n)			
Bronchodilators (e.g. ProAir)	0	13	
Taking >1 bronchodilator	0	4	
Antibiotics (oral or inhaled)	0	14	
Inhaled (e.g. Tobramycin)	0	9	

Data are mean±SEM or as n. FEV_1_, forced expiratory volume in one second; FEF_25-75_ = forced expiratory flow at 25.0 – 75.0 % of forced vital capacity

### Protocol overview

Prior to arrival for study participation, individuals were asked to refrain from caffeine and exercise for 24 h before arrival for study testing. Upon arrival to the environmentally controlled physiology laboratory for baseline testing, participants were fitted with a 12-lead electrocardiogram (Marquette Electronics, Milwaukee, WI) to monitor heart rate (HR) and rhythm. A single venous puncture blood draw (2 × 5 mL samples) occurred to assess hemoglobin and renal function. Following blood sampling standard pulmonary function testing (*i.e.* flow volume loop: forced expiratory volume at 25 to 75 % of FVC (FEF25-75), forced expiratory volume at 25 % of FVC (FEF25), forced expiratory volume at 50 % of FVC (FEF50), and forced expiratory volume at 75 % of FVC (FEF75)) in a seated upright position, using a Medical Graphics CPFS system spirometer (Medical Graphics, St. Paul, MN) according to the guidelines of the American Thoracic Society were performed in triplicate to ensure all measures fell within 10 % of each attempt [23]. Remaining in the upright position, participants had systolic (SBP) and diastolic (DBP) pressures assessed using manual sphygmomanometry. Assessment of Q followed using the acetylene rebreathe technique as described previously and in brief below [24]. All cardiovascular measurements were performed at baseline and at both 30-minutes (30min) and 60-minutes (60min) following administration of albuterol (described below). For each measurement time point we calculated the following: mean arterial blood pressure (MAP) = DBP + 1/3(SBP–DBP); body mass index = weight/height^2^; body surface area (BSA)= $\sqrt{\left(\mathrm{height}\kern0.2em \times \kern0.2em \mathrm{weight}\kern0.2em /\kern0.2em 3600\right)}$; SVR=(MAP/Q)•80; SV=Q/HR; cardiac power (CP)=Q•MAP; stroke work (SW)=SV•MAP; and reserve (delta=30- or 60-minutes minus baseline; or, percentage change=baseline to 30min or 60min) [25–27]. All cardiac hemodynamic measurements and SVR were also indexed separately to BSA: CP_I_, SW_I_, SV_I_, Q_I_, and SVR_I_.

Although not a primary aim of this study, to assess overall functional capacity (*i.e.* peak VO_2_), participants performed a seated upright peak cycle ergometry test to volitional fatigue (symptom limited) as described and reported previously by our group [8]. This assessment occurred on a separate study visit that was separated by at least 48 h but no greater than 1 week prior to participation in the present study. Predicted peak VO_2_ values were calculated according to Hansen et al. [28].

### Administration of albuterol

Following baseline testing and a brief rest period, participants were instructed to complete inhalation of the selective β_2_AR agonist albuterol (albuterol sulfate 2.5 mg diluted in 3.0 mL of normal saline) using a mouthpiece connected to a nebulizer (Power Neb2 nebulizer, Port Washington, NY) in a seated upright position. Using the mouthpiece—nebulizer system, individuals were instructed to breathe in calmly, deeply, and evenly for about 5-15 minutes until mist stopped forming in the nebulizer chamber. At 30min and 60min following albuterol administration, blood pressures were assessed in addition to Q in a similar manner to baseline.

### Measurement of cardiac output

In a seated upright position, participants breathed into a non-rebreathing technician controlled pneumatic switching Y-valve that was connected to a pneumotachometer and mass spectrometer. The inspiratory port of the switching valve allowed for rapid operator controlled switching between breathing room air or from a 5.0 L anesthesia rebreathe bag containing 1575 mL of test gas (0.65 % acetylene [C_2_H_2_], 9.0 % helium [He], 55.0 % nitrogen, and 35.0 % O_2_ as previously described [24].

Each rebreathe measurement period consisted of 8-10 consecutive breaths set at a cadence of 32 breaths/min using a metronome. During each rebreathe period, individuals were instructed to nearly empty the bag with each inspiration while the mass spectrometer was used to collect serial measurement of gas concentrations starting at end-expiration of the first breath during the rebreathe period which enabled rapid calculations of Q [24]. Acetylene disappears in the blood according to the rate at which pulmonary blood flow occurs, and therefore Q is calculated from the slope of the exponential disappearance of acetylene with respect to the insoluble gas He [24, 29, 30].

### Statistical analysis

All parametric data are presented as means ± standard error mean (SEM). The data were distributed normally. Homogeneity of variance of data was confirmed using Levene’s test. For comparisons of participant characteristics, Wilcoxon rank-sum tests were performed for variables except gender, which was compared using a χ^2^ test. Two-way ANOVA with repeated measures was used to test for the effect of participant and repeated measurement of hemodynamics. In the event of a significant F-test statistic, post-hoc testing with a Bonferroni correction was used. In addition, post-hoc *Cohen’s d* effect sizes (small=0.2; medium= 0.5; and large=0.8) were calculated to estimate the magnitude of effects for differences between groups at each level of time for cardiac hemodynamic and SVR indices [31, 32]. The alpha level was set at 0.05 to determine two-tailed statistical significance. All computations were made using SAS statistical software v.9.4 (SAS Institute Inc., Cary, NC).

## Results

### Study population

Baseline participant characteristics are presented in Table 1. Although controls did not have significantly higher body weight or body mass index compared to CF, none of the controls had evidence of nutritional deficiency. Although the absolute dose of albuterol was identical for all participants, because of the low body weight in individuals with CF, the dose of albuterol standardized for body weight resulted in a higher dose in CF compared to controls. Compared to controls, CF demonstrated lower functional capacity as indicated by a lower VO_2peak_ and percentage achieved of predicted VO_2peak_. As expected, spirometry tests prior to albuterol inhalation indicated pulmonary airway function was normal in controls but not in CF.

### Heart rate and peripheral vascular hemodynamics

Reported in Table 2 are absolute measurements of HR, SBP, DBP, MAP, SVR, and SVR_I_ assessed at baseline and at 30 and 60 min following inhalation of albuterol in participants. No significant interaction was observed for any test. Fixed effects from ANOVA models for participant (CF and controls) and condition (baseline, 30 min, and 60 min) were F(1, 47) = 10.91, p = 0.002, and F(2, 96) = 4.73, p = 0.011 for SVR, respectively; and, F(1, 47) = 18.13, p<0.0001, and F(2, 96) = 3.61, p = 0.0308 for SVR_I_, respectively.

**Table 2:** Heart rate and peripheral vascular function

	Minutes after albuterol		
	Baseline	30 min	60 min
**Variable**			
Heart rate (beats/min)			
CF	88±4	92±4	92±4
CTL	74±2*	77±2*	76±2*
Systolic blood pressure (mm Hg)			
CF	104±2	104±2	103±2
CTL	109±2	107±2	108±2
Diastolic blood pressure (mm Hg)			
CF	70±3	67±3	68±3
CTL	72±2	68±1	70±1
Mean arterial pressure (mm Hg)			
CF	82±2	76±3	80±2
CTL	84±1	81±1	83±1
SVR (dynes·sec/cm^5^)			
CF	1415±94	1276±96	1337±106
CTL	1301±60†	1095±50	1044±50*
SVR_I_ (dynes·sec/cm^5^/m^2^)			
CF	837±61	756±63	794±72
CTL	720±39^†^	603±30*	577±31*

Data presented as means±SEM. *CF* cystic fibrosis (*n*=18), *CTL* healthy individuals (*n*=30), *SVR* systemic vascular resistance, *SVR*_*I*_, systemic vascular resistance index; *CF vs. CTL, *p*<0.05; ^†^baseline vs. 60 min, *p*<0.05

At baseline, modest tachycardia was present in CF versus controls; although, all other variables were similar between groups. Differences in HR between groups at rest persisted to 30min (p<0.05), which was accompanied by higher SVR_I_ in CF compared to controls (p<0.05). Differences between groups at 30min persisted to 60min, which included significance for HR, SVR, and SVR_I_. *Cohen’s d* effect sizes between CF and controls for SVR and SVR_I_ progressively increased from baseline (0.31 and 0.50) to 30min (0.54 and 0.73) to 60min (0.82 and 0.93), respectively, suggesting that the differences between groups could be largely attributable to CF disease.

Within group differences comparing baseline to 30 or 60 min following albuterol showed no significant differences for any metric in CF. In contrast, at baseline, both SVR and SVR_I_ were significantly higher compared to 60min in controls, suggesting an influence of albuterol on increasing systemic vascular conductivity and permeability in controls.

### Cardiac hemodynamics

Presented in Table 3 are traditional measures of cardiac hemodynamics including Q, Q_I_, SV, and SV_I_, in addition to measures of cardiac pumping capability (*i.e.* CP, CP_I_, SW, and SW_I_) [25, 27]. No significant interaction effects were observed. From baseline to 60min, differences between groups in cardiac hemodynamics progressively increased, which were mainly due lack of increases in CF. This suggested that there was an influence of albuterol inhalation on increasing cardiac hemodynamics and arterial pressure generation in controls but not in CF. Increased effect sizes from baseline to 60 min following albuterol inhalation suggested that the differences between groups for cardiac hemodynamics were likely due to null responses in CF (Table 3).

**Table 3:** Cardiac hemodynamics

	Minutes after albuterol		
	Baseline	30 min	60 min
Variable			
Cardiac output (L/min)			
*F(1, 47)=15.72, p=0.0002; F(2, 96)=4.02, p=0.021*			
CF	4.9±0.3 (0.38)	5.2±0.3 (0.72)	5.2±0.4 (0.88)
CTL	5.5±0.3^†^	6.2±0.3*	6.7±0.3*
Cardiac index (L/min/m^2^)			
*F(1, 47)=6.11, p=0.0172; F(2, 96)=4.29, p=0.0165*			
CF	2.9±0.2 (0.06)	3.0±0.2 (0.45)	3.1±0.2 (0.64)
CTL	3.0±0.1^†^	3.4±0.1	3.7±0.2*
Stroke volume (mL)			
*F(1, 47)=27.79, p<0.0001; F(2, 96)=2.01, p=0.139*			
CF	59±5 (0.76)	62±6 (0.82)	60±6 (1.15)
CTL	74±3*^†^	82±5*	89±5*
Stroke volume index (mL/m^2^)			
*F(1, 47)=20.74, p<0.0001; F(2, 96)=2.28, p=0.108*			
CF	34±3 (0.66)	36±3 (0.68)	35±3 (1.01)
CTL	41±2*^†^	45±3*	49±3*
Cardiac power (L/min∙mm Hg)			
*F(1, 47)=20.90, p<0.0001; F(2, 96)=2.49, p=0.088*			
CF	401±27 (0.46)	386±23 (0.90)	415±29 (0.96)
CTL	466±27^†^	505±26*	554±27*
Cardiac power index (L/min∙mm Hg/m^2^)			
*F(1, 47)=13.14, p=0.0007; F(2, 96)=3.12, p=0.048*			
CF	234±15 (0.26)	225±11 (0.76)	242±16 (0.81)
CTL	252±13^†^	275±13*	301±13*
Stroke work (mL∙mm Hg)			
*F(1, 47)=35.98, p<0.001; F(2, 96)=1.55, p=0.218*			
CF	4786±384 (0.80)	4464±331 (1.09)	4763±463 (1.21)
CTL	6269±345*^†^	6610±401*	7334±405*
Stroke work index (mL∙mm Hg/m^2^)			
*F(1, 47)=32.59, p<0.001; F(2, 96)=1.92, p=0.1530*			
CF	2707±184 (0.77)	2567±147 (1.02)	2741±234 (1.09)
CTL	3423±176*^†^	3630±217*	4024±217*

Data presented as means±SEM. *CF* cystic fibrosis (*n*=18); *CTL* healthy individuals (*n*=30); Contained within parentheses are effect sizes (*Cohen’s d*) between CF and CTL; F-statistics from two-way ANOVA with repeated measures for fixed effects shown for participant (CF and CTL) and condition (baseline, 30 min, 60 min), respectively; *CF vs. CTL, *p*<0.05; ^†^baseline vs. 60 min, *p*<0.05

### Cardiac and peripheral hemodynamic reserve

**Fig. 1:**
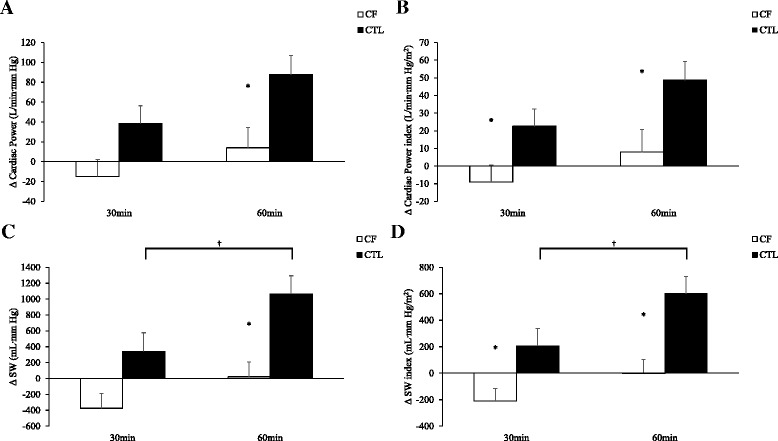
Data presented as means ± standard error mean. Cystic fibrosis (CF, *n*=18); healthy controls (CTL, *n*=30). Cardiac hemodynamic reserves calculated as the delta (Δ) at 30 or 60 minutes following albuterol inhalation minus baseline. Panels A and B, show reserves of cardiac power or cardiac power indexed for body surface areas (BSA) in individuals with CF versus CTL. Panels C and D, show reserves of stroke work (SW) or SW indexed for BSA in individuals with CF versus CTL. *CF versus CTL, *p*<0.05; ^†^Within group, *p*<0.05

**Fig. 2:**
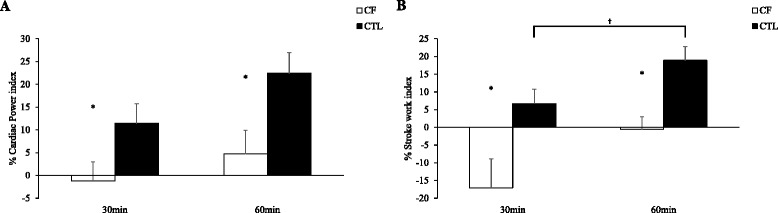
Data presented as means ± standard error mean. Cystic fibrosis (CF, *n*=18); healthy controls (CTL, 10.1186/s12931-015-0270-y *n*=30). Cardiac hemodynamic reserves calculated as a percentage change (%) from baseline to 30 or 60 minutes following albuterol inhalation. Panel A, shows cardiac power reserve indexed to body surface area (BSA) in individuals with CF versus CTL. Panel B, shows stroke work reserve indexed to BSA in individuals with CF versus CTL. *CF versus CTL, *p*<0.05; ^†^Within group, *p*<0.05

**Fig. 3:**
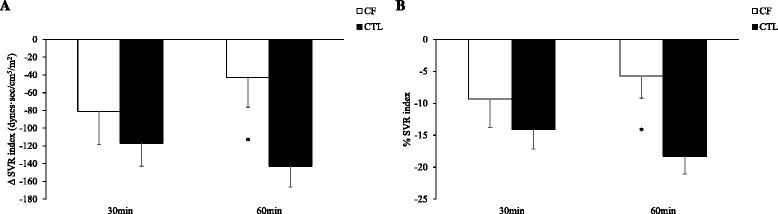
Data presented as means ± standard error mean. Cystic fibrosis (CF, *n*=18); healthy controls (CTL, *n*=30). Panel A, systemic vascular resistance (SVR) reserve indexed to body surface area was calculated as delta (Δ) at 30 or 60 minutes following albuterol inhalation minus baseline in individuals with CF versus CTL. Panel B, SVR index calculated as the percentage change (%) from baseline to 30 or 60 minutes following albuterol inhalation in individuals with CF versus CTL. *CF versus CTL, *p*<0.05

Presented in Figs. 1, 2 and 3 are CP, CP_I_, SW, SW_I_, SVR, and SVR_I_ reserves measured as differences between baseline and 30min or 60min following albuterol inhalation in participants.

We report in Fig. 1 (a–d), absolute differences (∆) between baseline and 30min or 60min for CP, CP_I_, SW, and SW_I_ following albuterol inhalation in CF and controls. In Panel A, *Cohen’s d* effect sizes between CF and controls for ΔCP were 0.62 at 30min and 0.77 at 60 min. In Panel B, *Cohen’s d* effect sizes between groups for ΔCP_I_ were 0.66 at 30 min and 0.73 at 60 min. In Panel C, *Cohen’s d* effect sizes between groups for ΔSW were 0.67 at 30 min and 0.99 at 60 min. In Panel D, *Cohen’s d* effect sizes between groups for ΔSW_I_ were 0.71 at 30 min and 1.01 at 60 min. The effect sizes at 30 min and to a greater extent at 60 min suggested a strong influence of CF disease on cardiac hemodynamic reserve responses to albuterol.

In Fig. 2 (a and b), we show percentage (%) change from baseline to 30min or 60min for CP_I_ and SW_I_ following inhalation of albuterol in CF and controls. Consistent with Fig. 1 (a–d) at 30 min in CF, there was a profound decrease in %SW_I_ at 30min following albuterol inhalation in CF. In Panel A, *Cohen’s d* effect sizes between CF and controls for %CP_I_ were 0.60 at 30 min and 0.75 at 60 min. In Panel B, *Cohen’s d* effect sizes between CF and controls for %SW_I_ were 0.81 at 30 min and 1.05 at 60 min. Observations in Fig. 2 (a and b) were consistent with Fig. 1 (a–d) for absolute differences.

Lastly, Fig. 3 (a and b) shows Δ and % change, respectively, for SVR_I_ from baseline to 30 min or 60 min following inhalation of albuterol in participants. In Panel A, *Cohen’s d* effect sizes between CF and controls for ΔSVR_I_ were 0.24 at 30 min and 0.74 at 60 min. In Panel B, *Cohen’s d* effect sizes between CF and controls for %SVR_I_ were 0.27 at 30 min and 0.84 at 60 min. These effect sizes suggested that there was a strong influence of CF disease on the attenuated SVR_I_ response to albuterol at 60 min and to a lesser extent at 30 min.

Overall, cardiac and SVR reserves were consistent with absolute measures presented in Tables 2 and 3, suggesting individuals with CF did not demonstrate normal cardiac and peripheral hemodynamic reserve responses to the β_2_AR selective-agonist albuterol.

## Discussion

In this study we present novel observations which suggest that individuals with CF demonstrate attenuated cardiac and peripheral hemodynamic responses to acute inhalation of the β_2_AR selective-agonist albuterol. The present findings support the hypothesis that mechanistic pathways closely associated with impaired β_2_AR function are likely determinants of cardiac and peripheral vascular dysfunction in CF. In this context, we show, 1) although absolute measures of baseline SV and SW are significantly lower in CF compared to controls, this attenuation in CF persists even with β_2_AR stimulation via albuterol. Whereas, in response to albuterol inhalation, healthy individuals in the present study and previously [22] demonstrate significant increases in cardiac hemodynamics; 2) similar to null cardiac hemodynamic responses to albuterol inhalation, vascular conductivity and permeability indicated by absolute measures of SVR are not responsive to albuterol inhalation in CF. In contrast, and consistent with previous observations from our group [22], reductions in SVR at 60 min following albuterol in healthy individuals suggest that improvements in peripheral vascular conductivity are related to β_2_AR stimulation; 3) differences in cardiac and peripheral hemodynamics from baseline to 30 or 60 min following albuterol inhalation suggest that individuals with CF are nearly entirely absent of cardiac and peripheral hemodynamic reserves, whereas healthy individuals are not; and finally, 4) although we did not assess exercise responses in the present study, these data are consistent with previous observations which clearly demonstrate that individuals with CF have attenuated cardiac hemodynamic responses to exercise and, hence, impaired cardiac reserve [9, 11]. Therefore, because arterial pressure is highly blood-flow mediated in the presence of intact cardiac hemodynamic function [33, 34], a decrease in vasodilatory reserve accompanied by an attenuated rise in cardiac hemodynamics could likely explain negligible SVR decreases following albuterol inhalation in CF.

Recent observations in animal models of CF suggest that abnormal cardiac structure, rhythmicity, and contractility in CF are associated with absent and/or dysfunctional CFTR [35, 36]. This is important since mutations in genes coding for CFTR are the cause of CF, and deranged or absent expression of CFTR is responsible for mechanisms leading to pulmonary dysfunction in individuals with this disease [2, 7–10].

The role of CFTR in contributing to the maintenance of electrochemical ion balance extends beyond the pulmonary system, whereby functional CFTR mediate protein kinase A-stimulated Cl^−^ currents in myocardial tissue, which is a relationship suggested to act to protect against ischemia or rhythm disturbances [37]. However, despite a well-known presence of β_2_AR in myocardial tissue, which are critically important in compensating for loss of β_1_AR and contractility, particularly in the condition of heart failure [20]; to date, it remains unclear what role impaired β_2_AR have in mediating cardiac dysfunction in CF. In this context, this is a noteworthy gap in knowledge as it is suggested that β_2_AR may have an important role in regulating CFTR-mediated Cl^−^ secretion and intracellular Ca^2+^ in the pulmonary system [38–41]. Thus, despite a more clear understanding of relationships between CFTR and β_2_AR pathways in pulmonary function [40, 42], any link CFTR shares with β_2_AR responsiveness in cardiac and peripheral vascular tissue is currently unknown in CF disease. This is an important question to answer since individually, or in an integrated capacity, mechanisms of CFTR, β_2_AR, and global cardiovascular system function appear related and markedly impaired in individuals with CF.

Although yet to be fully elucidated, it has been observed that alpha-1 adrenergic receptor (α_1_AR) sensitivity may be augmented in CF [43], which could lead to abnormal decreases in vasodilatory reserve. A heightened α_1_AR effector response of coronary and peripheral arteries to selective adrenergic sensitization could exacerbate a state of attenuated β_2_AR function. Hyperexcitability of α_1_AR in parallel with blunted systemic β_2_AR activity could lead to attenuated vasodilatory reserve and, hence, a negligible drop in SVR following albuterol inhalation. Therefore, because both α_1_- and β_2_-AR are highly involved in the regulation of Ca^2+^ flux that is essential for normal excitability and excitation-contraction coupling in cardiac myocytes and relaxation in smooth muscle [44–47], paradoxical differences in α_1_- and β_2_-AR receptor responsiveness may have critical consequences on cardiac and peripheral vascular function in CF. Dual, but imbalanced, sensitization of α_1_- and β_2_-AR via albuterol in cardiac and vascular tissue, where α_1_AR sensitivity is heightened and β_2_AR responsiveness is blunted could result in a critical reduction in intracellular Ca^2+^, thereby leading to an impaired rise in myocardial contractility that is accompanied by decreased relaxation of smooth muscle [46].

As such, because of shared pathways which contribute to the influence of β_2_AR on CFTR-mediated Cl^−^ regulation [38–42]; impaired β_2_AR, CFTR, Cl^−^, and Ca^2+^ mechanisms likely play a critical role in the cardiac and peripheral vascular hemodynamic health of individuals with CF. The present observations demonstrate clinical relevance as they suggest that β_2_AR selective-agonist therapy intended to alleviate signs and symptoms of pulmonary dysfunction in CF may be contraindicated in certain individuals. β_2_AR selective-agonists such as albuterol could augment effector responses of α_1_AR stimulation in the presence of diminished β_2_AR function. This could potentially contribute to an exacerbation of Ca^2+^ and Cl^−^ channel dysregulation leading to impaired excitation-contraction coupling in cardiac myocytes and uncoupling in smooth muscle and, hence, cardiac dysfunction concurrent with attenuations in vascular permeability and conductivity in individuals with CF. Moreover, as β_1_AR outnumber β_2_AR in a ~3:1 ratio in cardiac tissue [20], while also demonstrating sensitivity (lesser) to albuterol [48], acute increases in HR may indeed be a potential consequence of β_2_AR selective-agonist therapy, thereby causing unanticipated tachycardia. Although, consistent with pharmacokinetic studies of albuterol in CF and in healthy individuals [49], tachycardia that individuals with CF demonstrated in the present study was not likely due to the influences of albuterol, since elevated HR at rest persisted, but did not increase further at 30- and 60-minutes following albuterol.

These data present novel evidence for the potential role of cardiac and smooth muscle tissue β_2_AR pathways as targets for novel therapies in the management of signs and symptoms associated with global cardiovascular dysfunction in individuals with CF.

### Limitations

The present study was a cross-sectional design and pharmacokinetic testing was not conducted to assess systemic absorption of albuterol. However, both albuterol and method of delivery utilized in the present study have been previously demonstrated to elicit pulmonary function responses in CF and in healthy individuals and, therefore, we do not believe another confounding factor was causal for hemodynamic responses measured at 30 and 60 min following albuterol inhalation [13, 14, 22]. Moreover, detailed pharmacokinetic studies of inhaled albuterol in CF and healthy individuals show that despite similar inhaled body weight standardized dose concentrations of albuterol, albuterol absorption in the systemic circulation is markedly higher in CF compared to controls (area under the curve=1051.6±256.2 vs. 277.5±91.5 ng×sec/mL, respectively) [49]. Also, in comparison to systemic absorption of albuterol, it is estimated that only ~10% of inhaled albuterol is absorbed in pulmonary tissue [49], suggesting that in the presence of intact β_2_AR, there is a stronger likelihood of response at the systemic level compared to the pulmonary system. Lastly, the present study did not specifically test β_1_AR or α_1_AR function, nor intracellular Ca^2+^ or Cl^−^ as a result of albuterol inhalation in participants. Therefore, our hypotheses regarding the relationships between these factors warrants further study of the integrated roles of CFTR, α_1_AR, β_1,2_AR, Ca^2+^, Cl^−^, and cardiac and vascular function in individuals with CF.

## Conclusions

The major findings of this study suggests that individuals with CF do not demonstrate cardiac and peripheral vascular hemodynamic responsiveness to the short-acting β_2_AR selective-agonist albuterol that mirror normal responses of healthy individuals. We suggest that null cardiac and peripheral vascular hemodynamic responses to acute albuterol inhalation in individuals with CF are likely due to deranged β_2_AR function, closely accompanied by impaired CFTR and related pathways leading to augmented Cl^−^ and Ca^2+^ dysregulation in cardiac and vascular smooth muscle.

## Abbreviations

α_1_AR: alpha-1 adrenergic receptor
β_1_AR: beta-1 adrenergic receptor
β_2_AR: beta-2 adrenergic receptor
BSA: Body surface area
Ca^2+^: Calcium
CF: Cystic fibrosis
CFTR: Cystic fibrosis conductance regulator
Cl^−^: Chloride
CP: Cardiac power
DBP: Diastolic blood pressure
ENaC: Epithelial Na^+^ channel
FEF25-75: Forced expiratory volume at 25 to 75 % of FVC
FEF25: Forced expiratory volume at 25 % of FVC
FEF50: Forced expiratory volume at 50 % of FVC
FEF75: FEV_1_: forced expiratory volume in 1-second; forced expiratory volume at 75 % of FVC
FVC: Forced vital capacity
HR: Heart rate
MAP: Mean arterial pressure
Na^+^: Sodium
Q: Cardiac output
SBP: Systolic blood pressure
SD: Standard deviation
SV: Stroke volume
SVR: Systemic vascular resistance
SW: Stroke work
VO_2_: Oxygen uptake
